# Comparison of Laparoscopic Steerable Instruments Performed by Expert Surgeons and Novices

**DOI:** 10.3390/vetsci7030135

**Published:** 2020-09-15

**Authors:** Luca Lacitignola, Rodrigo Trisciuzzi, Annarita Imperante, Laura Fracassi, Alberto Maria Crovace, Francesco Staffieri

**Affiliations:** 1 Dipartimento dell’Emergenza e dei Trapianti di Organi (DETO), Sezione di Cliniche Veterinarie e P.A, Università degli Studi di Bari “Aldo Moro”, 70010 Bari, Italy; l.fracassi123@gmail.com (L.F.); francesco.staffieri@uniba.it (F.S.); 2 Dottorato di Ricerca in “Trapianti di Tessuti ed Organi e Terapie Cellulari”, Dipartimento dell’Emergenza e dei Trapianti di Organi (DETO), Università degli Studi di Bari “Aldo Moro”, 70100 Bari, Italy; rodrigo.trisciuzzi@uniba.it (R.T.); annarita.imperante@uniba.it (A.I.); 3 Scuola di Bioscienze e Medicina Veterinaria, Università di Camerino, 62024 Matelica, Italy; alberto.crovace@unicam.it

**Keywords:** laparoscopy, steerable instruments, learning curve

## Abstract

As an alternative to the surgical robot, some medical companies have engineered new steerable devices that mimic the robot’s capacities. This study aimed to assess how steerable instruments ameliorate the efficacy of suturing in comparison with the traditional instrument, and a combination instruments, performed by experienced and novice surgeons. The study was performed by three experienced surgeons and three novice surgeons. The instruments employed were divided into three surgical sets: two steerable dissectors; one steerable dissector and one straight needle; two straight needle holders. The study supervisor recorded the total time for the procedure, the number of bites completed, the time for each bite, and the quality of the procedure. In our study, we found consistent data demonstrating that experienced laparoscopists completed the prescribed suture pattern with more bites in less time than novices. The use of two steerable instruments was more time consuming than standard straight instruments, but a combination of instruments was significantly less time consuming, as was the use of two straight needle holders. This result was even observed in novice surgeons. Combining a steerable instrument with a traditional straight needle holder provided more advantages in this study.

## 1. Introduction

Surgical robots were introduced into human surgical procedures in the late 1990s to overcome the limits of conventional laparoscopy, including difficulties with dexterity and challenges [1].

Among the various features of robotic surgical systems, the stereoscopic vision, wristed instruments, and tremor filtration are considered to improve performance on laparoscopic tasks [2].

Robotic surgery was currently largely employed in many surgical specialties, in particular in urologic procedures [3]. Robotic surgery offers a faster learning curve, especially regarding suture techniques [4,5,6], with the presumably shared benefits of laparoscopy including less perioperative morbidity, improved visibility and precision, and faster recovery. The disadvantages of the robotic approach include the cost of the device [7] and the fact that they are not currently available in veterinary medicine.

In the last fifteen years, there has been substantial interest in creating devices that can provide some of the advantages of surgical robots, but at a lower cost. For example, steering devices include the articulated tip of an instrument that mimics a traditional laparoscopic instrument and is less like a robot [8]. The steerable tip enlarges the instrument’s workspace and allows the surgeon to reach structures that were otherwise inaccessible before [9]. Many studies have demonstrated the feasibility and advantages of steerable devices in clinical applications [2,4,8,9,10,11,12,13,14,15,16]. Such instruments are also beneficial for single-port laparoscopic surgery, which seeks to reduce invasiveness and trauma to the patient. Steerable instruments are listed as an essential method for reducing instrument crowding and providing triangulation at the surgical site [17].

The aim of this study was to assess potential benefits of the steerable instruments alone and in combination with a traditional straight needle holder in basic suture tasks executed by experienced and novice veterinary surgeons. Our hypothesis was that steerable tools could shorten learning curves and improve the quality of the suturing.

## 2. Materials and Methods

### 2.1. Operators

The study was performed by 3 experienced veterinary surgeons (Group Expert) with at least 50 laparoscopic procedures performed per year and 3 novice veterinary surgeons (Group Novice) with no experience in laparoscopic surgery. 

### 2.2. Surgical Setting

A Customised Phantom consisting in Polyurethane foam covered by elastic bandage tape was introduced into a laparoscopic trainer box (Ethicon, Cincinnati, OH, USA). With an n° 23 scalpel blade, a 6 cm cut was performed on the bandage layer of the phantom, at 90° to the operator. The laparoscopic trainer was covered with a towel to avoid the view inside the box. Three 5 mm trocars were used to access the box. A 30° angle of vision laparoscope (HOPKINS II, Karl Storz GMBH & Co. KG, Tuttlingen, Germany) was introduced into the central portal, and two instruments were introduced into the right and left portals.

**Figure 1:**
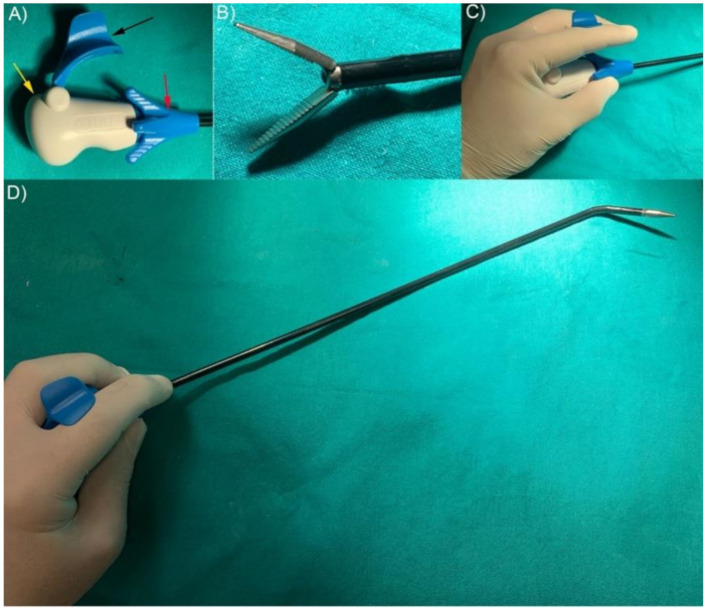
Laproflex dissector. (**A**) Instrument handpiece (yellow arrow). Jaw’s opening/closing activator (black arrow). Long axis 360° rotation roll (red arrow). (**B**) Laproflex dissector grip design. (**C**) Handling Laproflex. (**D**) Steering Laproflex is activated by the wrist.

**Figure 2:**
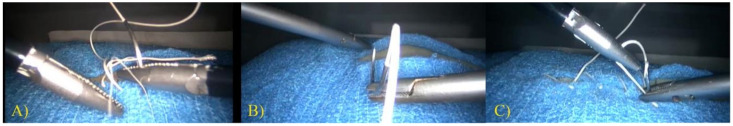
Surgical sets. (**A**) Laproflex. (**B**) Straight needle holder. (**C**) Combination of Laproflex and straight needle holder.

The Instruments employed were divided into three surgical sets: two 5 mm Laproflex dissectors (DEAM, Amsterdam, The Netherlands) (Laproflex Group) (Figure 1A–D; Figure 2A), of which one was used as needle holder (off-label use as it is not intended for that purpose); 2 Straight needle holders (Straight Group; Ethicon, Cincinnati, OH, USA; Figure 2B); 1 Laproflex dissector + 1 Straight needle holder (Mix Group; Figure 2C). The suture material employed for the suturing task was 3-0 USP polyglactin 910), with a 26 mm 3/8 circle reverse cutting needle.

A prerecorded instruction video of all the exercises was shown at the start of the program, and all participants were allowed to manipulate the instruments for 10 min in order to familiarize themselves with the handling and activation of the instruments’ movements. Each operator performed a simple continuous pattern suture with one surgical knot at the first bite and then a maximum of 8 loops 4–5 mm apart from each other, starting on the left of the cutting line. Each session was repeated (one session per day) 3 times for the experts, and 5 times for the novices. A maximum of 45 min was allowed for each operator to complete the suturing task.

All procedures were video recorded, and the study supervisor, blinded to the operator, recorded the total time for the procedure and the number of bites completed. The time for each bite (including the knot tying time) was obtained by dividing the total time by the total number of bites performed.

The quality of the procedure was quantified by applying a modified Objective Structure Assessment of Technical Skills (mOSATS) laparoscopic skills evaluation scale, considering the followings parameters: (1) load needle perpendicular to needle driver; (2) choke needle 1/2 to 2/3 from needle tip; (3) place needle at a 90 degree angle to tissue; (4) pull the suture through to establish short free end; (5) suture placed accurately on target. The surgeon’s knot quality was evaluated according to the following requirements: (6) knot laid flat without air knots; (7) short free end maintained; (8) appropriate tissue reapproximation without strangulation; (9) good use of both hands to facilitate knot tying. The supervisor assigned 1 for positive evaluation and 0 for negative; the sum of the previous score assigned was registered for each trial (max 9 points).

### 2.3. Statistical Analysis

Minitab^®^ 19 Statistical Software (Suturentry, UK) Data were summarized as the mean ± standard deviation (SD), Inerquartile range (IQR), and 95% confidence interval (95% CI). The Shapiro–Wilk test was used to assess normality before evaluation. One-way analysis of variance was used to test for differences between the groups for normal distributed data. A Tukey–Kramer test was used for post hoc analysis. A Wilcoxon signed-rank/Kruskal–Wallis test was used to test nonparametric data. Power analysis was calculated posthoc for time × bite. To evaluate the learning curve, the R^2^ was calculated on the basis of time × bite vs. the number of attempts. Values of *p* < 0.05 were considered significant.

## 3. Results

The expert group completed all procedures in the prescribed time. Conversely, the novice surgeons completed the procedures only five times, abandoning the procedure before completing the minimum number of bites planned or exceeding the maximum time available. The actual power analysis for time per bite vs. the operator group was >90%.

**Figure 3:**
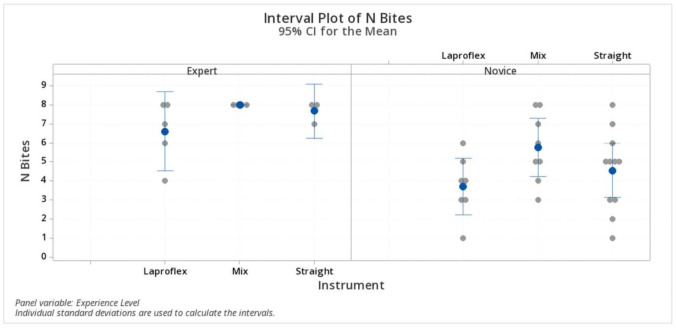
Number of bites. The difference was statistically significant (*p* < 0.05) between the surgeons’ groups but not for the type of instruments employed.

The mean number of bites in the expert group was 6.6 ± 1.6 (IQR 5.00–8.00; 95% CI 5.43–7.76) bites for the Laproflex instruments, 8 ± 0.00 (IQR 8.00–8.00; 95% CI 6.7–9.29) bites for the combination of instruments, and 7.667 ± 0.577 (IQR 7.00–8.00; 95% CI 6.16–9.16) for the straight instruments. In the novice group, the number of bites completed with the Laproflex instruments was 3.71 ± 1.60 (IQR 4.00–5.00; 95% CI 2.22–5.20), 5.75 ± 1.83 (IQR 4.225–7.75; 95% CI 4.35–7.14) with the combination of instruments, and 4.45 ± 2.11 (IQR 3.00–6.00; 95% CI 3.35–5.73) for the straight instruments. The difference was statistically significant (*p* < 0.05) between the surgeons’ groups (considering all surgical sets together), but specific comparison analysis performed between the groups of surgeons for each surgical set was not significant (*p* > 0.05) for the type of instruments employed (Figure 3).

**Figure 4:**
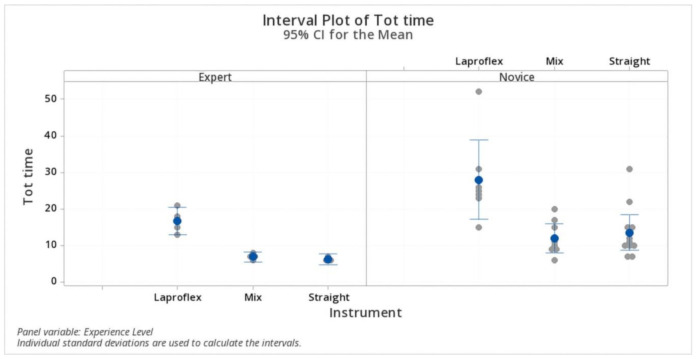
Total time. The Laproflex instruments were significantly more time consuming than the other instrument sets in both the expert and novice group. The level of experience (although with a lower tendency in the expert group) did not influence the total time.

The mean total time elapsed by the experienced surgeons was 16.80 ± 3 min (IQR 14.00–19.50; 95% CI 14.68–18.92) with the Laproflex instruments, 7 ± 0.81 (IQR 6.25–7.75; 95% CI 4.63–9.36) with the combination of instruments, and 6.33 ± 0.577 (IQR 6.00–7.00; 95% CI 3.59–9.06) with the straight instruments. Conversely, for the novice surgeons group, the mean total time was 28.00 ± 11.60 min (IQR 23.00–30.00; 95% CI 21.73–34.27) with the Laproflex instruments, 12.13 ± 4.73 (IQR 9.00–16.50; 95% CI 6.26–17.99) with the combination of instruments, and 13.64 ± 7.16 (IQR 10.00–15.00; 95% CI 8.64–18.64) with the straight instruments. The Laproflex instruments were significantly (*p* < 0.05) more time consuming than the other instrument sets in both the expert and novice group. The comparison of the level of experience (although with a lower tendency in the expert group) did not influence the total time significantly (*p* > 0.05) (Figure 4).

**Figure 5:**
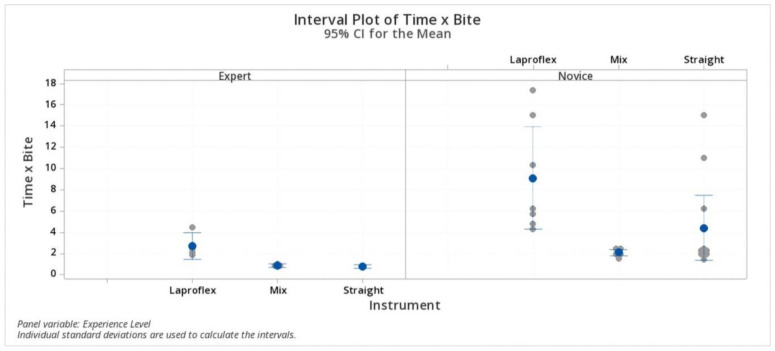
Time × bite. Experienced surgeons had significantly lower time × bite with all instrument sets.

In the expert group, the calculated time elapsed to complete a single bite, including the knot tying, was 2.72 ± 1.04 min (IQR 1.91–3.56; 95% CI 2.01–3.42) with the Laproflex instruments, 0.87 ± 0.1 min (IQR 0.78–0.96; 95% CI 0.08–1.66) with the combination of instruments, and 0.82 ± 0.06 min (IQR 0.75–0.87; 95% CI −0.08–1.73) with the straight instruments. The Laproflex instrument set was significantly higher (*p* < 0.05) in both the surgeons’ groups. In the novice group, sutures performed with Laproflex instruments took 9.11 ± 5.24 min (IQR 4.80–15.00; 95% CI 5.98–12.25), 2.093 ± 0.32 min (IQR 1.85–2.43; 95% CI −0.81–5.02) with the combination of instruments, and 4.41 ± 4.53 min (IQR 2.00–6.20; 95% CI 1.91–6.91) with the straight instruments. In this group, the combination of instruments was significantly less time consuming than the other instrument sets. Experienced surgeons had significantly lower time × bite with all instrument sets (Figure 5).

Regression analysis for time × bite vs. the order of procedures fitted the curve at 66.2% significance in the novice surgeons’ group with Laproflex instruments. No other regression analysis demonstrated significant levels (*p* > 0.05). 

**Figure 6:**
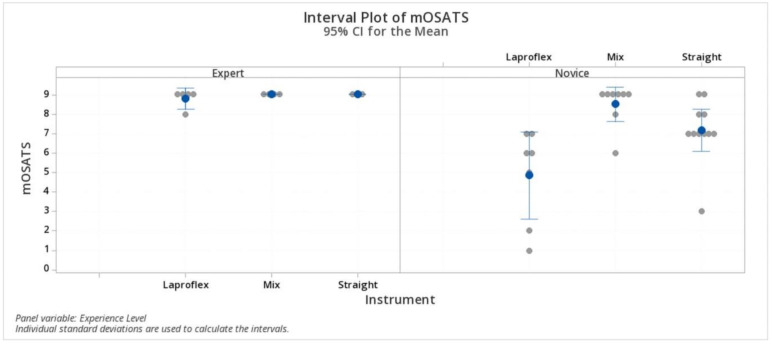
Modified OSATS score. Statistically significant differences were detected in the novice group using Laproflex instruments but not with the straight instruments or the combination of instruments. Statistically significant differences (*p* < 0.05).

The modified OSATS score was 8.8 ± 0.44 (IQR 8.5–9; 95% CI 8.49–9.1) in the expert group with Laproflex instruments, 9 ± 0 (IQR 9.0–9.0; 95% CI 8.66–9.33) with the straight instruments, and 9 ± 0 (IQR 9.0–9.0; 95% CI 8.61–9.38) with the combination of instruments. In the novice group, the mean score was 4.8 ± 2.4 (IQR 2.0–7.0; 95% CI 3.5–6.2) with the Laproflex instruments, 7.1 ± 1.6 (IQR 7.0–8.0; 95% CI 7.23–9.76) with the straight instruments, and 8.5 ± 1.06 (IQR 8.2–9.0; 95% CI 6.1–8.25) with the combination of instruments. The modified OSATS score was statistically significantly lower (*p* < 0.05) in the novice group using Laproflex instruments but not with the straight instruments or the combination of instruments, while in the experienced surgeons, no differences were detected. (Figure 6).

## 4. Discussion

This study aimed to assess the role of steerable laparoscopic instruments in performing basic suture tasks by experienced laparoscopists and novice surgeons, comparing straight instruments and a combination of a steerable instrument and a straight needle holder. 

In our study, we found consistent data demonstrating that experienced laparoscopists completed the prescribed suture pattern with more bites in less time than the novices. Despite our hypothesis, the use of two steerable instruments was more time consuming than the standard straight instruments, but a combination of instruments was significantly less time consuming, as was the use of two straight needle holders. This result was even observed in novice surgeons, who performed each bite in less time than with straight and two steerable instruments.

We found that the off-label use of the Laproflex instrument as a needle holder, although it is ergonomic and designed for intuitive movement, was cumbersome. The Laproflex dissector jaw does not have a grip designed for holding the needle and does not have a locking system to block the needle into the jaws during movements. We observed needle drops during bite deployments and slippage of the suture during knot tying, in particular in the novice group, but also in the first trials in the expert group. Moreover, for both operator groups enrolled in this study, accurate suture placement on the target was difficult, because the instrument bent at the shaft during the maneuvers and the force applied was not efficient in terms of completing bites or passing the needle through the tissue. It is likely that the stiffness of the distal bending section should be improved to avoid loss of energy through the shaft; in fact, when using rigid instruments, the distance from the handle to the tip is constant, improving the transmission of the force needed to place the needle [18]. Moreover, a rigid shaft follows the wrist supination, improving the efficacy of forces. 

In this study, both surgeons’ groups found the Laproflex surgical set difficult to pass the needle through the phantom material. In particular, the main exertion was in maintaining the jaws closed to hold the needle, but needle rotation, slipping, and dropping were not avoided. Even during this off-label use, in one attempt, the handle of the instrument was broken by a novice surgeon. In our study, the number of bites performed was significantly higher in experienced surgeons compared to novices. This result was mainly attributed to the abandonment of the procedure by novices. Loss of instrumental control provided fatigue and disappointment leading to the abandonment of procedures in the novices [19]. The physical and mental workload for laparoscopic surgeons might be counterproductive for the operative time and quality of hand-sewn anastomoses or other demanding procedures [20].

The use of two Laproflex instruments was significantly more time consuming than the other instrument sets in both the expert and novice group. However, analyzing the time elapsed to complete a bite, we found that regardless of the instrument set used to perform tasks, experienced surgeons had a significantly lower time to complete a single bite. 

The mOSATS score assessed the quality of the tasks performed [21] and represents an objective tool to evaluate surgical skills acquired by surgeons in training [22,23,24]. In this study, combining a steerable instrument with a traditional straight needle holder provided more advantages. In fact, experienced and novice laparoscopists improved as regards the time and quality of the suture. The main benefit when employing the steerable instruments was during knot tying, because the steerable instrument facilitated the creation of the loops for making the surgical knot. Moreover, because they allow for more degrees of freedom, steerable instruments provided help in needle positioning on the straight needle holder jaws and also in grasping the edge of the tissue for a proper and precise bite. With steerable instruments, the distal part is mostly positioned perpendicular to the viewing direction [18]. The two additional degrees of freedom of tip deflection and rotation were useful for difficult access angles to the operative field [13].

The regression analysis showed that the experience level influenced the learning curve. Interestingly, Novice surgeons improved in terms of the time and quality of the suture more quickly than the experienced surgeons, but the skilled surgeons were more efficient with any technique performed. We can speculate that experienced surgeons were more familiar with the traditional techniques and developed less adaption in changing suture techniques. In fact, in a laparoscopic gastro-jejunal anastomosis study, the use of a novel deflectable instrument in an ex vivo model demonstrated a fast learning curve for all participants, but the learning curve of novices was even more rapid [20]. In that study, even though the anastomosis time of the novices came closer to that of trained surgeons after a number of trials, the statistical analysis revealed that the average suturing times and the complete anastomosis times were still expectably different when comparing the fifth trial of the untrained to experienced participants.

We suppose that a more extended period of training combining these two instruments could provide evidence of better results.

A limitation of this study was the artificial environment of the suture phantom used. We suppose that a cadaveric study would provide a better assessment of the tissue and feedback sensation, more closely mimicking the in vivo condition. However, an accurate simulation should provide an environment as close as possible to the reality, mimic visual-space and real-time characteristics of the procedure, and provide realistic haptic feedback [25]. From dedicated studies, it is known that robotic surgery requires a distinct learning curve different from laparoscopic and open surgery [26]. As this might be similar for steerable instruments, a comparison between steerable hand-held instruments and robotic instruments should be conducted on validated tasks to identify accurate differences in learning curves [27]. Training was demonstrated to be useful for trained and untrained subjects as only a limited number of tasks were necessary to improve both quality and time [28,29,30]. This experimental study showed a decrease in the required suturing time in both groups, which clearly indicates a fast learning effect when switching from standard laparoscopic instruments to a combination with steerable instruments. In this study, only three experienced surgeons and five novices were recruited to perform surgical tasks. We are aware that this could represent a limitation of the study, and enlarging the number of investigators will improve the power of the study.

Competent handling of Laproflex instruments can be acquired quickly even if with no previous experience in laparoscopic suturing, but the use of the dissector as a needle holder influenced our results. We suggest re-engineering the steerable instruments in terms of grip design, locking system, and the shaft stiffness of the instruments for needle holder function. 

In conclusion, although the steerable instrument has more functions to be actuated as compared to conventional instruments, it required more training to learn to control those additional functions in this study. More studies should be performed in cadavers or in vivo in a full laparoscopic environment to confirm the results of this study and study real advantages of the steerable laparoscopic instruments.
